# Risk and resilience correlates of reading among adolescents with language-based learning disabilities during COVID-19

**DOI:** 10.1007/s11145-022-10361-8

**Published:** 2022-11-11

**Authors:** Rebecca A. Marks, Rachel T. Norton, Laura Mesite, Annie B. Fox, Joanna A. Christodoulou

**Affiliations:** 1 Department of Communication Sciences and Disorders, MGH Institute of Health Professions, 36 First Avenue, Boston, MA 02129 USA; 2 School of Healthcare Leadership, MGH Institute of Health Professions, 36 First Avenue, Boston, MA 02129 USA

**Keywords:** Learning disabilities, LBLD, Reading, Socio-emotional skills, Resilience, COVID-19

## Abstract

Students with language-based learning disabilities (LBLD) can face elevated socio-emotional well-being challenges in addition to literacy challenges. We examined the prevalence of risk and resilience factors among adolescents with LBLD (*N* = 93), ages 16–18, and the association with reading performance during the COVID-19 pandemic. Data were collected at the start and end of the first fully remote academic year of COVID-19 (2020–2021). Participants completed standardized word and text reading measures, as well as self-report surveys of executive functions (EF), and socio-emotional skills associated with resilience (grit, growth mindset, self-management, self-efficacy, and social awareness) or risk (anxiety, depression, COVID-19 related PTSD, and perceived COVID-19 impact). Survey data at the start of the school year (Time 1) captured three underlying factors associated with socioemotional risk, socioemotional resilience, and regulation (i.e., EF). Path analyses revealed that students’ Time 2 oral reading scores were significantly and uniquely predicted by socioemotional resilience, even when controlling for word-level reading at Time 1. Socioemotional risk, EF, and perceived COVID-19 impact were not directly related to Time 2 oral reading scores; however, students’ resilience mediated the associations between risk and reading outcomes. These results demonstrate that adolescents’ mental health concerns, self-regulatory ability, and socioemotional resilience were all associated with their experiences of the COVID-19-related stress. However, despite the high-risk context of the pandemic, and socio-emotional challenges faced by students with LBLD, our findings indicate that resilience directly predicts end-of-year reading outcomes and mediates the impact of socioemotional risk on achievement.

**Supplementary Information:**

The online version contains supplementary material available at 10.1007/s11145-022-10361-8.

Experiences during the COVID-19 pandemic negatively impacted academic skills and mental health for many students (Ellis et al., 2020; Racine et al., 2021), particularly adolescents at risk for academic or socio-emotional challenges (Baschenis et al., 2021; Bosch et al., 2022; Korpa et al., 2021). The present study examines the impact of the COVID-19 pandemic with a focus on adolescents with language-based learning disabilities (LBLD), who are at heightened risk for academic challenges and comorbid psychiatric or mental health difficulties. Guided by a cumulative risk and resilience model of reading impairment (Catts & Petscher, 2022), we examine the associations between socio-emotional risk and resilience factors, adolescents’ experiences of the COVID-19 pandemic, and their reading outcomes after a year of remote schooling. We center this study around connected text reading, which is both an area of challenge for students with LBLD, and of particular importance for content area learning, as well as for college and career readiness (Paige, 2011; Rasinski et al., 2017; Royer et al., 1990).

Language-based learning disabilities are difficulties in using and/or understanding oral and/or written language. LBLDs typically fall under the category of specific learning disabilities in school contexts (Individuals with Disabilities Education Act, 2004), and are considered a neurodevelopmental disorder termed specific learning disorder in diagnostic contexts (DSM-5; American Psychiatric Association, 2013). Students with LBLD may have a range of disabilities, including developmental language disorder, dyslexia, and reading comprehension impairment, and may experience difficulties with skills such as decoding, encoding, oral reading fluency, orthographic processing, narrative comprehension and production, syntax, and grammar (Colozzo et al., 2011; Kida et al., 2016).

Reading comprehension and reading fluency are two major areas of concern for students with LBLD, whose reading rate may be labored and slow with difficulty accessing the meaning of text (National Joint Committee on Learning Disabilities, 2008; Snow & Biancarosa, 2003). The challenge of reading and understanding connected text is that it is not a singular skill, but rather a complex activity dependent on a range of knowledge and skills (Catts, 2022). By adolescence, readers are expected to be able to read with sufficient skill to extract and analyze content knowledge from text. Students with LBLD, however, come to the task of decoding and understanding text with additional, longstanding processing challenges of oral or written language. In addition, students with LBLD also face elevated concern for anxiety, depression, and related psycho-social vulnerabilities (Hendren et al., 2018). Intersecting biological, environmental, and psychosocial factors can contribute a varied spectrum of reading abilities and longitudinal outcomes for this population (Yu et al., 2018).

The COVID-19 pandemic brought environmental changes that may have disproportionately impacted literacy development in students with learning disabilities. In particular, the pandemic may have generated or increased disparities between students receiving special education services and their peers (Yüksel et al., 2021), a potential consequence of the swift change to home isolation and remote learning. One study conducted with university students in Poland reported that students with reading difficulties experienced higher stress levels and worse academic achievement during the pandemic than their peers who did not report having reading difficulties (Zawadka et al., 2021). Another study of Italian children found that, although the majority of participants improved in their reading over the course of lockdown, children with dyslexia showed a learning trajectory that was less steep (i.e., slower rate) than predicted (Baschenis et al., 2021). These learning challenges may be further compounded by socio-emotional experiences. During the COVID-19 pandemic, adolescents reported decreased mental health compared to pre-pandemic levels (Racine et al., 2021) and reduced self-confidence, happiness, and social connection (Margolius et al., 2020).

Little is known, however, about how the COVID-19 context, *in conjunction* with other socio-emotional risk and resilience factors, may have affected literacy outcomes in students with LBLD. Given the compounded risk of having a learning disability during a time of school disruption, it is critically important we examine the risk and resilience factors that may mitigate or exacerbate the effects of adverse experiences. For students with reading difficulties in particular, strong executive function and socio-emotional skills can be sources of resilience that may attenuate student challenges (Haft, 2016), while trauma, stress, and executive function deficits can be risk factors that may exacerbate vulnerabilities (Catts & Petscher, 2022). We review specific socio-emotional risks in the context of the COVID-19 pandemic before turning to resilience factors.

## Risk factors

### COVID-19 stress

The COVID-19 pandemic created a global health emergency with serious risk to physical and mental health. Although adolescents were not at higher risk for severe infection, they may have been a subgroup more susceptible to negative mental health consequences (Guo et al., 2020). Fear of COVID-19 infection or exposure may increase symptoms of psychological stress, while prolonged risk of COVID-19 effects presents a continuous risk for negative effects on mental health (Ellis et al., 2020; Guo et al., 2020).

Of particular importance for adolescents, the COVID-19 pandemic resulted in a rapid transition to remote learning and increased isolation, particularly during the 2020–2021 school year. In a study of 3,300 adolescents and young adults, Margolius et al. (2020) found that changes brought on by COVID-19 resulted in considerably less time spent on academic activities, decreases in sleep due to emotional and cognitive stressors, a loss in self-confidence, and reported unhappiness, depression, and reduced connection to teachers and peers. Another study of high school students found that nearly a quarter of students reported pandemic-related concerns along with increased anxiety, depression, and loneliness (Gazamarian et al., 2021). Although both studies highlighted disproportionate negative impacts of the pandemic by demographic group (i.e., gender, race, socio-economic status), neither study specifically examined adolescents with learning disabilities (Gazamarian et al., 2021; Margolius et al., 2020), a population likely to be even more vulnerable to the pandemic’s negative effects (Kuhfeld et al., 2020).

### Generalized anxiety and depression

Clinically significant symptoms of anxiety and depression can negatively affect the academic and social experiences of adolescents (Magson et al., 2021). Adolescents with LBLD may be at particularly high risk of elevated mental health difficulties, related to both their learning difficulties and the context of the COVID-19 pandemic. First, students with learning disabilities may be at particular risk for comorbid anxiety and/or depression (e.g., Nelson & Harwood, 2011). Children with reading disabilities generally exhibit greater anxiety and worse depressive symptoms than their typically developing peers (Mammarella, 2016), and anxiety is negatively associated with academic performance in students with reading impairments over time (Hossain et al., 2021a). Similarly, adolescents (Giovagnoli et al., 2020) and college students (Ghisi et al., 2016) with dyslexia report increased anxiety and depression, which have also been associated with lower self-esteem. Second, evidence suggests increased levels of anxiety and/or depression in adolescents during the COVID-19 pandemic (De France et al., 2022; Magson et al., 2021; Racine et al., 2021), although findings are mixed. (For studies demonstrating lower levels of adolescent anxiety or depression during the pandemic, see Luthar et al., 2021a, 2021b.) Greater anxiety and depression during the pandemic was associated with online learning difficulties (Magson et al., 2021), concerns about academic performance (Luthar et al., 2021a), and more dramatic lifestyle changes associated with the pandemic (De France et al., 2022).

### Post-traumatic stress disorder

Post-traumatic stress disorder (PTSD) is characterized by dissociative-, anxiety-, or fear-related manifestations that arise after exposure to a traumatic event, or after being repeatedly exposed to details of the event (APA, 2013). While those directly affected by COVID-19 (i.e., infection, sickness, experiencing a family member’s death) are at risk for traumatic stress, the fear of COVID-19 infection or exposure can similarly create a risk of developing traumatic stress (Guo et al., 2020). Traumatic experiences in adolescent populations may involve single or multiple exposures to stressors, and have demonstrated adverse effects on cognitive, linguistic, and social-emotional domains (op den Kelder et al., 2017). In their risk and resilience model of dyslexia, Catts and Petscher (2022) suggest that experiences of trauma may confer cumulative risk related to reading difficulties. Independent of pandemic-related trauma, individuals with learning disabilities may already experience some school-based trauma related to their learning differences (Doyle & Mitchell, 2003).

## Resilience factors

In the face of the COVID-19 pandemic, it is critical to recognize the resilience factors that may buffer against poor mental health and academic outcomes. Resilience is typically understood as the capacity to successfully adjust to risk or adversity (Masten & Barnes, 2018). Although many factors can be understood under the umbrella of resilience, for the present study, we follow prior reading disability frameworks in distinguishing between *socioemotional resilience* and *cognitive resilience* (Catts & Petscher, 2022; Haft et al., 2016), though we acknowledge potential overlap.

### Socioemotional resilience

Students may demonstrate resilience via social and emotional domains. Children with well-developed core socio-emotional skills demonstrate awareness of their emotions, social interactions, and personal life goals, in addition to the ability to make responsible decisions related to those goals (Oberle et al., 2016). Specifically, social-emotional proficiency is imperative for academic success, as students navigate communication with peers and teachers, difficult situations, and decision-making opportunities that may affect outcomes in educational contexts and beyond (Zins et al., 2007).

Socio-emotional skills are thought to play a critical role in adolescent outcomes, particularly among those affected by traumatic experiences, as well as populations identified with specific learning difficulties (Hendren et al., 2018). On one hand, students with learning disabilities are more likely to have lower academic self-efficacy and endorse fixed beliefs about intelligence—maladaptive characteristics that are negatively associated with performance and achievement (Baird et al., 2009). On the other hand, students with learning difficulties who feel greater control over their own learning demonstrate better academic outcomes (Zheng et al., 2014). In a sample of reading impaired children and young adolescents, parent and teacher ratings of students’ resilience were positively related to academic performance (Hossain et al., 2021b). By building socio-emotional competencies, students may be better equipped to face challenges (Oberle et al., 2016), particularly those associated with reading difficulties (Haft et al., 2016). The current study therefore assesses numerous dimensions of socioemotional resilience (i.e., self-management, self-efficacy, social awareness, grit and growth mindset), and examines their association with mental health risk and COVID-19-related stress as well as reading outcomes.

Socio-emotional competencies may attenuate the associations between risk or negative stressors and academic outcomes. Resilience can be understood as a mediating process that allows some individuals to achieve positive outcomes despite the presence of risk. For instance, Chinese adolescents’ resilience mediated the effects of perceived stressful life events on their school adjustment, attenuating the negative impact of stress (Zhang et al., 2019). The COVID-19 pandemic presents a novel context of wide-reaching perceived risks and life stressors with which students must cope. We offer that socioemotional resilience may impact reading skills through numerous potential mechanisms, including through stress and attention systems. At the psychological level, socioemotional resilience may support students in reframing and responding to challenges effectively through underlying stress response regulation. Resilience may also help students to deploy and sustain their attention related to academic topics effectively, even in the context of environmental risk factors.

### Cognitive resilience

Cognitive factors can likewise play a role in attenuating the challenges of a learning disability and/or serve as compensatory skills. While many dimensions of both cognitive and linguistic processing can serve as resilience factors (Haft et al., 2016), we focus on executive functions in this study. Executive functions (EFs) are top-down cognitive processes that help with attention, emotion-regulation, problem-solving, impulse control, goal-oriented behavior, and self-management (op den Kelder et al., 2017). Broad EF domains include working memory, inhibition, and cognitive flexibility (Meiri et al., 2019). Strong EF (i.e., self-regulation) skills are protective factors associated with improved academic, health, and well-being outcomes in school-aged populations (Zins et al., 2007), whereas children with EF deficits may experience impulsivity, intensified emotional reactions, and difficulty with goal-directed behavior (op den Kelder et al., 2017).

EF is of particular interest for individuals with LBLD, including those with reading difficulties, because EFs are closely linked to underlying processes for reading (Church et al., 2019). EF in early-childhood is predictive of later reading abilities (Blankenship et al., 2019; Thompson et al., 2015), and high EF may help to differentiate between children at risk for dyslexia who do or do not go on to develop reading impairments (Eklund et al., 2013). One potential explanation is that high levels of EF may partially compensate for low decoding abilities to support higher level reading comprehension skills (Cirino et al., 2019). At the same time, however, reading difficulties frequently co-occur with EF deficits (Al Dahhan et al., 2022; Booth et al., 2010; Lonergan et al., 2019). EF can be considered simultaneously as a potential area of weakness for some LBLD students (Al Dahhan et al., 2022; Eklund et al., 2013) and a promising protective factor that may buffer against the adverse effects of major life stressors for others (Shields et al., 2017; Zhang et al., 2019).

## Current study

Most studies of interventions for RD and comorbid diagnoses such as anxiety and depression analyze co-occurring diagnoses individually, indicating a need for future work to address relationships among comorbid factors (Hendren et al., 2018). We present a study with an adolescent sample with LBLD, examining stress related to COVID-19, risk factors (i.e., post-traumatic stress disorder, anxiety, depression), resilience factors (i.e., social-emotional skills, executive functions), and performance on reading measures, to identify predictive relationships among variables, at the start and end of a fully remote school year during the COVID-19 pandemic.

## Method

### Participants

Ninety-three adolescents (63 male, 30 female) participated in this study. All participants were enrolled in grade 11 or 12 at a school serving students with learning disabilities located in the Northeast region of the United States. Participants’ age at Time 1 ranged from 16.08 to 18.92 years old (*M* = 17.37, *SD* = 0.68 years). Students all carried LBLD diagnoses; although specific diagnostic data is not available per participant, historically, over 90% of the enrollees at this school have been identified with a specific learning disability (SLD). Participant performance on this study’s measures (see Table 1) align with the common reading profile of LBLD students, with 87% performing below the average range (standard score of < 85 or scaled score of < 8) on at least one reading measure, 72% on at least two measures, and 60% on three or more.

**Table 1:** Percentage of students scoring at or below the age-normed mean score on T1 literacy measures

	≤ 85 (%)	≤ 90 (%)	≤ 100 (%)
Word reading	31.2	63.4	91.4
Pseudoword decoding	34.4	54.8	92.5
Oral reading quotient	58.1	80.6	97.8

Data were collected by trained school staff as standard of care in Fall 2020 (October through December) and Spring 2021 (April through June), during the first complete academic year of the COVID-19 pandemic. Deidentified data were provided for secondary data analysis in accordance with approved procedures by the Partners Human Research Committee Institutional Review Board.

## Measures

### Reading

Reading performance was measured at the word and connected text levels. Word level skills were indexed using untimed real word reading and untimed pseudoword reading subtests from the Kaufman Test of Educational Achievement, Third Edition (KTEA-3; Kaufman & Kaufman, 2014). Split-half reliability coefficients for these subtests range from 0.95 to 0.97 in 16–19 year olds (Kaufman & Kaufman, 2014).

Connected text reading performance was measured using the Gray Oral Reading Test, Fifth Edition (GORT-5; Wiederholt & Bryant, 2012). Age-normed standardized scores are used in all statistical analyses. Standardized scores for reading rate, accuracy, fluency, and comprehension are based on a mean of 10 and a standard deviation of 3, and the oral reading index is based on a mean of 100 and a standard deviation of 15. Internal reliability for the GORT-5 subtests range from 0.92 to 0.98 in 16–19 year olds (Wiederholt & Bryant, 2012).

### Executive function

EF was measured with the Behavior Rating Inventory of Executive Function, Second Edition (BRIEF-2) Self-Report (Gioia et al., 2015). This measure has strong internal consistency and reliability, with self-report reliability coefficients ranging from 0.71 to 0.97 (Hendrickson & McCrimmon, 2019). Three indices of self-regulation are reported. The Behavior Regulation Index includes inhibition (i.e., controlling impulsivity) and self-monitoring (i.e., monitoring one’s behavior in relation to others). The Emotion Regulation Index includes shifting (i.e., transitioning between situations) and emotional control (i.e., emotion regulation skills). The Cognitive Regulation Index includes task completion (i.e., efficiency when completing school tasks), working memory (i.e., holding task-relevant information in mind), and planning/organizing (i.e., goal setting and organizing goal-directed action). Higher BRIEF-2 scores are indicative of higher clinical concern for EF difficulties. Standardized scores, which are used in all analyses, are based on a mean of 50 and a standard deviation of 10; scores above the average range are considered mildly elevated (60–64), potentially clinically elevated (65–69), or clinically elevated (at or above 70).

### Socio-emotional resilience

Subtests derived from the Panorama Social-Emotional Learning Survey (Panorama Education, 2015) were used to evaluate participants’ social-emotional skills. Mean scores on 5-option Likert scales were used to quantify students’ grit, growth mindset, self-management, social awareness, and self-efficacy. Cronbach’s ɑ for each subtest ranged from 0.82 to 0.93, indicating good internal consistency reliability.

The Self-Efficacy subscale (5 items, ɑ = 0.91) asked participants to rate their confidence in their ability to achieve academic outcomes (e.g., “How confident are you that you can complete all the work that is assigned in your classes?”), ranging from 1 = “Not at all confident” to 5 = “Extremely confident.” The Self-Management subscale (10 questions, ɑ = 0.93) asked participants to consider their skill with self-regulation in the classroom within the past 30 days (e.g., “How often did you follow directions in class?”), ranging from 1 = “Almost never” to 5 = “Almost all the time.” The Grit subscale (5 questions, ɑ = 0.82) asked participants to respond to questions about their experience persevering through challenges, such as, “If you fail to reach an important goal, how likely are you to try again?” (1 = “Not at all likely” to 5 = “Extremely likely”). The Growth Mindset subscale (6 questions, ɑ = 0.86) asked participants to rate their belief that factors such as intelligence, effort, and talent can be changed or developed, ranging from 1 = “Not at all possible to change” to 5 = “Completely possible to change.” The Social Awareness subscale (8 questions, ɑ = 0.89) asked participants to consider their empathetic interactions with others within the past 30 days, such as “When others disagreed with you, how respectful were you of their views?” (1 = “Not at all respectful” to 5 = “Extremely respectful”).

### Risk factors

Risk factors included participants' perceived impact of the COVID-19 pandemic, as well as their risk of experiencing anxiety, depression, and PTSD symptomology. Cronbach’s ɑ for these subtests ranged from 0.83 to 0.96.

#### COVID-19 impact

A modified version of the COVID-19 Impact Scale was used to quantify self-reported experiences (Ellis et al., 2020). This 8-item scale was designed for adolescents, and invites responses regarding the pandemic’s impact on the school year, family finances, social relationships, and related concerns (i.e., “To what extent are you worried about how COVID-19 will impact you feeling connected to your friends?” and “How likely is it that you could become infected with the COVID-19 virus?”). The survey response options were modified from the original three-point scale to be: (1) Not at all, (2) A little bit, (3) Somewhat, (4) Quite a bit, and (5) A tremendous amount. Internal consistency reliability was ɑ = 0.83.

#### Anxiety and depression

The Pediatric Item Bank, PROMIS Emotional Distress Battery (PROMIS Health Organization, 2013a, 2013b) was used to measure anxiety (13 items, i.e., “My worries overwhelmed me”) and depression (14 items, i.e., “I felt that I had nothing to look forward to”). A 5-point Likert response scale was used, and raw scores were converted to T scores, in which higher values indicate higher risk across 4 categories: None to slight, Mild, Moderate, and Severe. Internal consistency reliability for the anxiety and depression scales were ɑ = 0.94 and ɑ = 0.96, respectively.

#### Post-traumatic stress symptoms

Following the COVID-19 Impact Scale, the UCLA Brief COVID-19 Screen for Child/Adolescent PTSD was administered (UCLA Brief Screen for Child/Adolescent PTSD, 2020). The 11-item scale assessed participants’ potential risk of PTSD symptoms related to the COVID-19 pandemic. Using a 5-point response scale (ɑ = 0.85), participants described how frequently during the past month they have had experiences like, “When something reminds me of what happened or is still happening, I get very upset, afraid, or sad.” Total scores were interpreted in four categories with higher scores indicating a higher risk for PTSD: No PTSD symptoms; Minimal PTSD symptoms; Mild PTSD symptoms; or Potential PTSD. This PTSD screener has strong internal consistency and reliability with subjects ages 7–18, and can discriminate between individuals who do vs. do not meet full PTSD diagnostic criteria (Kaplow et al., 2020).

### Analysis

To examine the associations between socio-emotional resilience, risk, and reading skill, we performed structural equation modeling in MPlus Version 8.5 (Muthén & Muthén, 1998–2017). Model fit was examined using the Chi square value and four goodness-of-fit indices: the comparative fit index (CFI) > 0.95, Tucker-Lewis index (TLI) > 0.95, root mean square error of approximation (RMSEA) < 0.06, and standardized root mean square residual (SRMR) < 0.08 (Kline 2015).

**Fig. 1:**
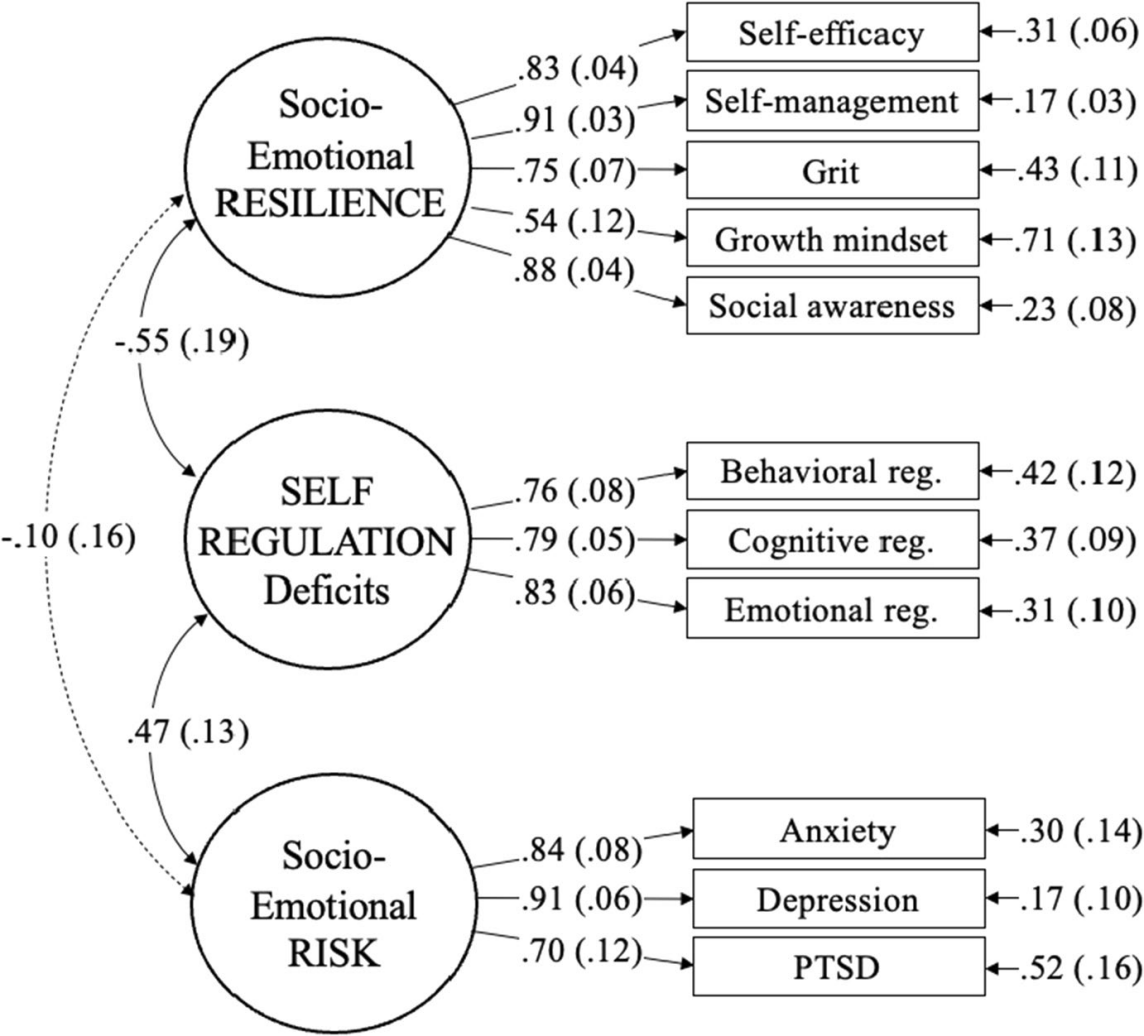
Factor analysis representing socio-emotional resilience, self-regulation deficits, and socio-emotional risk

First, we conducted an exploratory factor analysis (EFA) to examine the underlying latent structure of the risk, resilience, and regulation measures assessed in the fall (Time 1), allowing for two to four factors. The three factor model revealed theoretically principled underlying factors representing socioemotional resilience (all five subscales of the socioemotional learning survey), mental health risk (depression, anxiety and PTSD surveys), and self-regulation (all BRIEF-2 indicators), and was an adequate fit to our data (CFI = 0.96, TLI = 0.91, RMSEA = 0.09, SRMR = 0.05). Notably, students’ mean COVID-related stress did not load well onto any factor. The four factor model suggested a separate latent construct with COVID-related stress, growth mindset, and PTSD symptomatology as indicators. Although including this fourth factor improved statistical fit ($\chi$^2^_diff (4 FAC − 3 FAC)_(9) = 30.25, *p* < 0.001), it was not theoretically aligned with prior literature. We subsequently removed students’ mean COVID-related stress from the factor analysis, and ran a follow-up confirmatory factor analysis (CFA) with three factors using a maximum likelihood robust (MLR) estimator (Fig. 1; CFI = 0.94, TLI = 0.92, RMSEA = 0.08, SRMR = 0.07). Given the small sample size, we exported factor scores derived from this three factor CFA to represent risk, resilience, and self-regulation in subsequent path models. We then examined two competing models of the associations between these socio-emotional predictors at Time 1, as operationalized using these factor scores, and participants’ text-level reading skills in the spring (Time 2), measured using the GORT-5 Oral Reading Quotient. We controlled for age and word reading ability (KTEA-3 untimed real word reading) at Time 1.

## Results

### Descriptive statistics

#### Reading measures

Table 2 presents the descriptive statistics regarding participants word- and text-level reading skills as measured at Time 1. As expected, this sample’s reading difficulties are reflected in low average mean scores across word-level tasks and by below average mean scores across passage reading tasks.

**Table 2:** Descriptive statistics and correlations between variables at Time 1

	*M*	SD	Range	1	2	3	4	5	6	7	8	9	10	11	12	13	14	15	16	17
1. Behavior regulation^a^	52.59	9.06	39.00–85.00																	
2. Cognitive regulation^a^	56.25	10.11	38.00–85.00	0.63***																
3. Emotion regulation^a^	54.43	9.56	38.00–89.00	0.62***	0.65***															
4. Growth mindset^b^	3.49	1.10	1.00–5.00	− 0.18	− 0.16	− 0.21*														
5. Self-management^b^	4.13	0.81	1.00–5.00	− 0.43***	− 0.36***	− 0.37***	0.47***													
6. Self-efficacy^b^	3.60	0.99	1.00–5.00	− 0.43***	− 0.46***	− 0.43***	0.48***	0.75***												
7. Grit^b^	3.56	0.82	1.00–5.00	− 0.39***	− 0.34***	− 0.32***	0.49***	0.66***	0.69***											
8. Social awareness^b^	3.89	0.76	1.00–5.00	− 0.43***	− 0.33***	− 0.35***	0.46***	0.83***	0.70***	0.63***										
9. Anxiety^c^	44.50	11.64	32.30–88.00	0.13	0.26*	0.32**	0.04	− 0.05	− 0.12	0.09	0.06									
10. Depression^c^	48.35	12.61	31.70–86.60	0.22*	0.32***	0.47***	− 0.06	− 0.13	− 0.13	0.02	− 0.01	0.77***								
11. PTSD^d^	7.44	6.78	0.00–33.00	0.20*	0.34***	0.43***	0.09	− 0.22*	− 0.28**	− 0.02	− 0.15	0.60***	0.62***							
12. COVID stress^e^	2.32	0.81	1.00–4.25	0.12	0.17	0.24*	0.37***	0.17	0.08	0.14	0.16	0.34***	0.18	0.33***						
13. Word reading^f^	88.17	10.03	51.00–111.00	0.00	− 0.03	0.02	− 0.10	− 0.12	0.08	− 0.09	− 0.17	− 0.13	− 0.06	− 0.05	0.04					
14. Pseudoword reading^f^	88.79	8.82	66.00–119.00	0.04	− 0.11	− 0.00	− 0.03	− 0.03	0.04	− 0.07	− 0.04	− 0.10	− 0.03	− 0.03	− 0.06	0.53***				
15. Text reading fluency^g^	6.43	1.91	2.00–11.00	0.04	− 0.03	− 0.01	0.13	0.15	0.22*	.06	0.05	0.04	0.01	0.06	0.13	0.51***	0.47***			
16. Reading comprehension^g^	7.23	1.72	2.00–12.00	0.01	0.06	− 0.05	− 0.03	0.19	0.21*	0.10	0.08	0.01	− 0.07	− 0.13	0.07	0.44***	0.20*	0.60***		
17. T1 oral reading quotient^g^	82.72	8.67	65.00–105.00	0.03	0.02	− 0.03	0.05	0.19	0.24*	0.08	0.07	0.03	− 0.03	− 0.03	0.11	0.53***	0.38***	0.91***	0.88***	
18. T2 oral reading quotient^g^	82.92	10.27	57.00–107.00	− 0.01	− 0.03	− 0.02	0.11	0.18	0.25*	0.09	0.06	− 0.03	− 0.02	0.02	0.13	0.49***	0.36***	0.68***	0.68***	0.76***

*N* = 93. Bolded values are statistically significant: *p < 0.05, **p < 0.01, ***p < 0.001. T1 = Time 1, T2 = Time 2

^a^BRIEF-2 subscale, ^b^Panorama SEL survey subscale, ^c^PROMIS Emotional Distress Battery T-score, ^d^UCLA PTSD screen total symptoms, ^e^COVID-19 Impact scale mean, ^f^KTEA-3, ^g^GORT-5

#### Executive function

EF indices show that the majority of participants are in the “not elevated” category for each BRIEF-2 index. Conversely, clinically elevated scores range from 3.2% (emotional control and self-monitor) to 11.8% (working memory). Frequency statistics for each category across the behavior regulation, cognitive regulation, and emotion regulation indices are presented in Supplemental Table S1.

#### Risk factors

On average, participants reported that they were “a little bit” to “somewhat” concerned about the COVID-19 pandemic (*M* = 2.68, *SD* = 1.40), with 30.1% of participants reporting “quite a bit” or “a tremendous amount” of concern. Participants were similarly worried that the pandemic would impact their school year (*M* = 2.71, *SD* = 1.36), with 34.4% of participants reporting “quite a bit” or “tremendous” concern. Of the eight items on the COVID-19 Impact Scale, participants were least concerned that they themselves might become infected with the virus (M = 1.03, SD = 1.02). Means and standard deviations for each item are presented in Table 3.

**Table 3:** Descriptive statistics for survey items on COVID-19 Impact Scale

	*M*	SD
To what extent are you worried about how COVID-19 will impact your school year?	2.71	1.36
To what extent are you worried about how COVID-19 will impact your own and your family’s finances?	2.34	1.15
To what extent are you worried about how COVID-19 will impact your ability to keep up your reputation?	1.85	1.10
To what extent are you worried about how COVID-19 will impact you feeling connected to your friends?	2.38	1.20
To what extent are you concerned about the COVID-19 crisis?	2.68	1.40
How likely is it that you could become infected with the COVID-19 virus?	1.03	1.02
How likely is it that someone you know could become infected with the COVID-19 virus?	2.45	1.22
If you did become infected with COVID-19, to what extent are you concerned that you will be severely ill?	2.09	1.19

All items are on a 5-point Likert scale from 1 = “Not at all” to 5 = “Extremely worried/concerned/likely”

The majority of participants demonstrated minimal risk for COVID-related PTSD, anxiety or depression. Anxiety T scores indicated 79.6% None to slight, 7.5% Mild, 10.8% Moderate, and 2.2% Severe. Depression T scores indicated 68.8% None to slight, 14.0% Mild, 12.9% Moderate, and 4.3% Severe. Risk as measured by the PTSD survey yielded 10.8% No PTSD symptoms, 63.4% Minimal PTSD symptoms, 20.4% Mild PTSD symptoms, and 5.4% Potential PTSD.

### Associations between COVID-19 impact, risk, and resilience variables

Descriptive statistics and Pearson correlations between each of the risk and resilience measures at Time 1 are also presented in Table 2. Correlations indicate that greater perceived impact of the COVID-19 pandemic (i.e., COVID-19 stress) is associated with more difficulties in emotional control, higher risk of anxiety and COVID-19-related PTSD symptoms, as well as a tendency towards growth mindset.

### Measurement and structural models

**Fig. 2:**
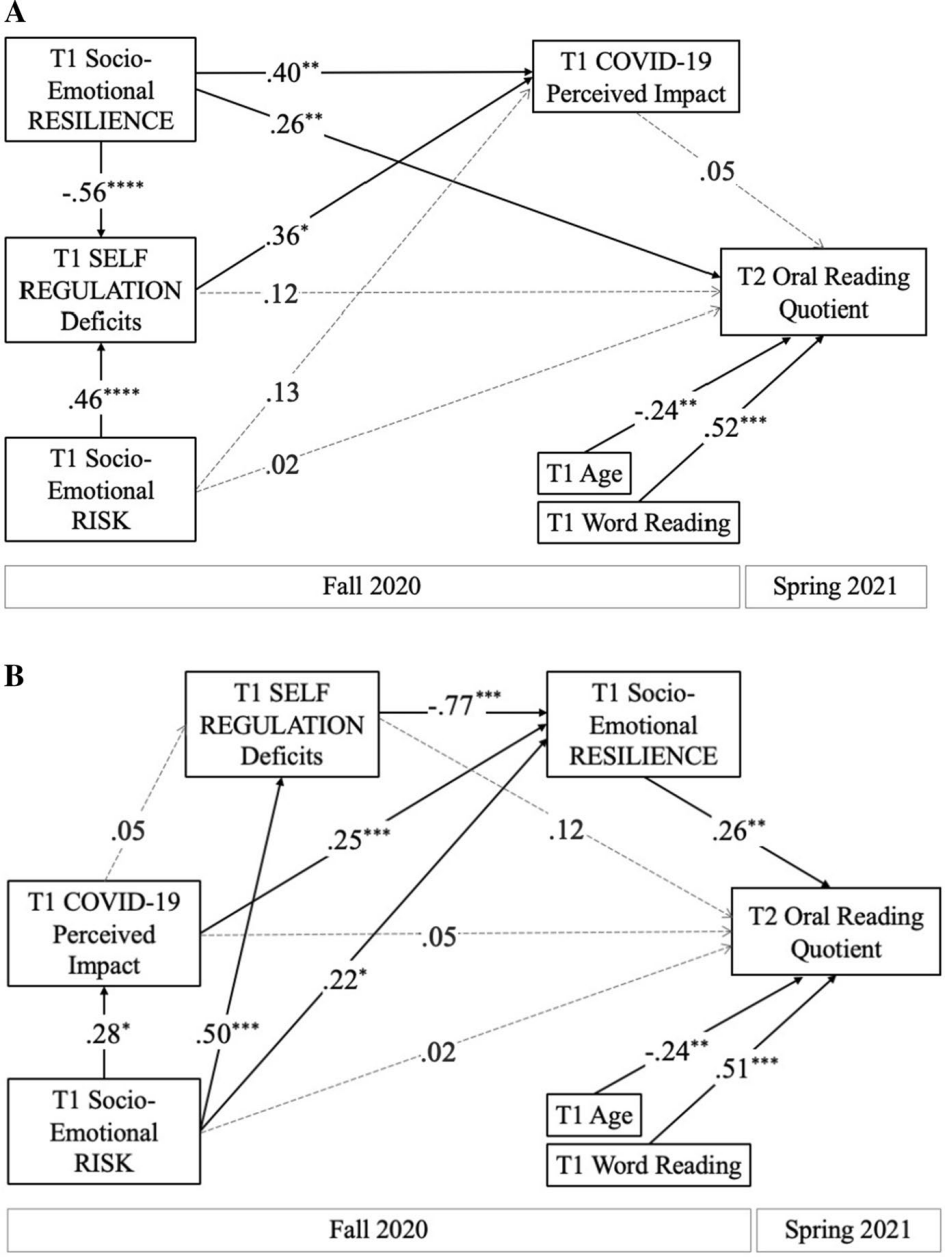
Path analysis models explaining variance in end-of-year oral reading skill in relation to students’ perceptions of the COVID-19 pandemic, as well as socio-emotional risk and resilience factors

We tested two path analysis models predicting participants’ oral text reading skills (GORT-5 Oral Reading Quotient) at the end of the school year (Time 2; Spring 2021). In the first model, we examined the direct effects of participants’ socioemotional resilience, risk, and self-regulation factor scores on an oral reading composite score, as well as the indirect effects of resilience, risk, and self-regulation through students’ perceived COVID-19 impact at Time 1 (see Fig. 2A). This model fit the data well (*X*^2^(4, *N* = 93) = 0.83, *p* = 0.935; CFI = 1.00, TLI = 1.00, RMSEA = 0.00, SRMR = 0.02) and explained 35% of variance in adolescents’ oral reading quotient. Model results revealed significant effects of both socioemotional risk and resilience on self-regulation deficits. The resilience and self-regulation factor scores were both significantly associated with students’ perceived COVID-19 impact at Time 1; however, only socioemotional resilience had a significant direct effect on Time 2 oral reading composite scores. These effects were replicated when using T2 reading comprehension as the dependent variable (see Supplement).

In an alternative model based on prior work by Zhang and colleagues (2019), we examined how self-regulation and resilience might mediate the effects of COVID-19 related stress and other risk factors on oral reading (Fig. 2B). Replicating the results of the first structural model, only socioemotional resilience had a significant direct effect on Time 2 oral reading. However, resilience also mediated indirect effects of self-regulation (β = − 0.20, *p* = 0.007) and perceived COVID-19 impact (β = 0.06, *p* = 0.043) on oral text reading composite scores (see Supplemental Table S3). There was also a significant indirect path from socioemotional risk to oral reading through both self-regulation and resilience (β = − 0.10, *p* = 0.016). This second model also fit the data well (*X*^2^ (6, *N* = 93) = 2.28, *p* = 0.892; CFI = 1.00, TLI = 1.00, RMSEA = 0.00, SRMR = 0.04) and explained 36% of the variance in students’ oral reading quotient. Again, the effects were replicated when using T2 reading comprehension as the dependent variable (see Supplement).

Finally, to investigate the components of the resilience factor that might be driving the consistent association with oral reading skill, we conducted post hoc partial correlations between each observed resilience variable and participants’ oral reading rate and accuracy (and a composite represented as fluency), and comprehension at Time 1 and Time 2. Reading skill was consistently associated with participants' self-management and self-efficacy (Table 4). Sensitivity analyses revealed that this pattern of associations was robust when controlling for age, sex, EF, and risk factors.

**Table 4:** Partial correlations between resilience factors at Time 1 and GORT-5 text-level reading skills

	Growth mindset	Self-management	Self-efficacy	Grit	Social awareness
T1 rate	0.18	0.23*	0.22*	0.17	0.15
T1 accuracy	0.18	0.25*	0.19	0.10	0.16
T1 fluency	0.21	0.25*	0.21*	0.12	0.16
T1 comprehension	0.01	0.27**	0.19	0.15	0.18
T1 oral reading quotient	0.13	0.30**	0.23*	0.16	0.19
T2 rate	0.15	0.20	0.22*	0.08	0.15
T2 accuracy	0.14	0.24*	0.15	0.08	0.12
T2 fluency	0.17	0.25*	0.23*	0.12	0.15
T2 comprehension	0.15	0.25*	0.21*	0.17	0.16
T2 oral reading quotient	0.18	0.27**	0.24*	0.16	0.17

*N* = 93, *p < 0.05, **p < 0.01. T1 = Time 1 (Fall 2020); T2 = Time 2 (Spring 2021). Text-level reading skills are measured by the Gray Oral Reading Test. Partial correlations control for KTEA-3 word recognition at T1

## Discussion

The current study examined factors associated with risk and resilience among adolescents with language-based learning disabilities (LBLD), and their relation to reading performance over a year of schooling during the COVID-19 pandemic. Across two path analysis models, students’ experiences of pandemic-related stress were associated with their socio-emotional resilience, mental health risk, and self-regulatory skills at the start of the school year (Fall 2020). End of year reading performance was positively predicted by socioemotional resilience, but was not directly associated with socioemotional risk or self-regulation. Adolescents’ mental health concerns, which are often heightened in LBLD populations, were associated with their stress related to the COVID-19 pandemic. However, despite the high-risk context of the pandemic, reading skills were directly predicted by resilience factors. Risk factors indirectly impacted reading ability, mediated by students’ resilience.

### Risk, resilience, and the experience of COVID-19-related stress

The onset of the COVID-19 pandemic disrupted home, school, and work environments as nations around the world implemented social distancing and lock-down measures. This dramatic contextual change was linked to poor mental health outcomes (Bosch et al., 2022; Li et al., 2021). Among our sample of 11th and 12th graders with LBLD, we found that greater COVID-19-related stress was correlated with PTSD symptomology and generalized anxiety, but not depression. The association with anxiety is consistent with prior studies, while the non-significant correlation between COVID-19 stress and depressive symptomology is unexpected. For instance, COVID-19 distress in a sample of Australian adolescents correlated with higher incidence of both generalized anxiety and depression, and was negatively associated with life satisfaction (Magson et al., 2021). Furthermore, pandemic-related distress moderated the change in mental health over time. Similarly, increased concern about the threat of COVID-19 was associated with higher incidence of anxiety, depression, and somatic symptoms among Chinese college students (Liu, Liu & Liu, 2020). Our findings are also consistent with studies that have reported increased prevalence of PTSD risk within the context of the COVID-19 pandemic (Sayed et al., 2021), and relationships between PTSD symptomology and anxiety and depression (Stewart et al., 2022).

Greater COVID-19-related stress was also correlated with increased difficulties with emotion regulation. Our path analyses shed light on the directionality of this association, revealing a significant path from the self-regulation deficits to COVID-19-related stress (Fig. 2A). In contrast, a second model (Fig. 2B) revealed no significant pathway from COVID-19 stress to self-regulation deficits, lending specificity to our correlational findings. This result is consistent with a longitudinal study revealing that pre-pandemic emotion dysregulation in U.S. teenagers was linked to greater mental health concerns during the pandemic (Breaux et al., 2021).

Interestingly, COVID-19 stress was also correlated with growth mindset. Put another way, adolescents who were more likely to endorse fixed beliefs about learning and intelligence (i.e., “It is not possible to change your level of intelligence”) experienced *lower* levels of COVID-related stress. This bivariate correlation is also reflected in the first path model, in which higher socioemotional resilience was associated with greater COVID-19 stress. The direction of this association stands in contrast to prior work: a large meta-analysis revealed *negative* associations between growth mindset and psychological distress, and a positive association between growth mindset and active coping mechanisms, for both adolescents and adults (Burnette et al., 2020). More specific to the COVID-19 context, the Health Belief Model (Rosenstock, Strecher & Becker, 1988) suggests that socio-emotional competencies such as self-efficacy should promote positive coping mechanisms. Higher self-efficacy has been shown to buffer the negative effects of health stressors and mental health problems in adults, through the mediator of COVID-19 risk perception (Zhou et al., 2021). Nevertheless, the association between greater socioemotional competence (particularly growth mindset) and greater perceived COVID-19 impact is apparent in our data.

We offer a few speculative hypotheses for this finding. One possible explanation is that adolescents—who have substantially less political and social capital through which to influence their surrounding environment compared to adults—may have had heightened awareness of the ways in which authority figures *could* have responded to pandemic risks, but may have failed to live up to adolescents’ hopes or expectations. Relatedly, adolescents may have experienced disempowerment or limited agency with reduced social engagement in school/home settings, which may have tempered associations between growth mindset and coping mechanisms. Additional research is needed to clarify how the associations between mindset, socioemotional competence, and risk perception may vary across contexts and individuals during COVID-19.

### Socioemotional resilience mediates risk and predicts oral reading skill over time

In their model of cumulative risk and resilience, Catts and Petscher (2021) suggest that socioemotional and cognitive resilience factors may buffer against the burden of reading difficulties. In the present study, we demonstrate a positive direct effect of socioemotional resilience at Time 1 on oral reading skill at Time 2. In contrast, we observed no direct effects of self-regulation deficits, mental health risk, or COVID-19-related stress on reading over time. Our second model shows that self-regulation, COVID-19 stress, and mental health risk are all *indirectly* associated with reading skill, mediated by adolescents’ socioemotional competence. Post-hoc correlations suggest that the two strongest socioemotional predictors of LBLD students’ oral reading skills were *self-efficacy* and *self-management.*

These findings contribute to the limited research connecting socioemotional resilience to reading achievement, particularly in adolescents, and provide unique insights into the role of resilience for students with LBLD. We extend prior work demonstrating that socioemotional resilience mediates the effects of stressful events on school adjustment among adolescents (Kim et al., 2018; Zhang et al., 2019). Results demonstrate that resilience may buffer the negative effects of COVID-19-related stress, mental health risk, and EF deficits on reading. We also find, consistent with prior research, that higher scores on EF and self-regulation is associated with increased resilience (Davidovich et al., 2016; Zhang et al., 2019). Notably, we find no direct effect of EF on end of year oral reading composite scores. The inclusion of socioemotional risk and resilience factors, or the overlap between these factors and our EF measures, may have led to contrasting findings with prior work on younger students, demonstrating direct associations between EF and reading skill (e.g., Cirino et al., 2019). However, little is currently known about the role of self-regulation in oral reading or reading comprehension in adolescents. It is possible that the associations between cognition and academic achievement are less pronounced in older compared to younger learners (e.g., Ferrer et al., 2007), or that EF was sufficiently represented in the socioemotional resilience questions indirectly.

Our findings extend the notion that socioemotional competence is positively related to academic adjustment more broadly in students with learning disabilities (Haft et al., 2016; Hossain et al., 2021b; Zheng et al., 2014) by connecting resilience directly to reading ability. There has been limited work to date with struggling learners that directly links socioemotional skills to specific domains of student achievement, and most extant research focused on the role of grit and/or growth mindset in younger children (Credé et al., 2017; Hossain et al., 2021b; Petscher et al., 2017; Sisk et al., 2018).

The present study’s findings suggest that in older adolescent readers with LBLD, self-efficacy and self-management may be associated with oral passage reading skill (fluency and comprehension). These findings extend prior work suggesting that self-efficacy is associated with reading comprehension skill in early adolescence. For instance, self-efficacy was positively correlated with, but did not uniquely predict reading comprehension above word reading in 7th grade struggling readers (Klauda & Guthrie, 2015). Similarly, while self-efficacy predicted initial reading comprehension skill in 6th graders, growth mindset (but not self-efficacy) predicted growth in reading (Cho et al., 2021). To our knowledge, our study provides early evidence linking both self-efficacy and self-management to reading achievement in older readers (11th and 12th grade) with language-based learning disabilities.

One challenge when interpreting our results in conjunction with prior work is the variability in how socioemotional learning, self-efficacy, and self-regulation are operationalized. However, two recent studies with high school students begin to clarify these associations. First, Fairless and colleagues (2021) examined the associations between socioemotional skills, environmental supports, and achievement in a large sample of high-risk U.S. high schoolers. Academic achievement across subjects was positively correlated with students’ socioemotional skills and self-efficacy. In a regression model, self-efficacy was a strong positive predictor of achievement, whereas socioemotional learning (conceptualized in terms of task management, peer relationships, and self-regulation skill) did not predict unique variance (Fairless et al., 2021). Like Fairless and colleagues (2021), we find positive effects of self-efficacy on achievement, though we operationalize socioemotional skills differently. A second study with Canadian high schoolers demonstrated an association between an SEL intervention, which included heart rate monitoring and direct instruction in behavior regulation, and reading comprehension test performance specifically (McLeod & Boyes, 2021). The intervention group’s test-taking self-efficacy remained stable over the course of the study—in contrast to a decline in self-efficacy in the control group—and they exhibited reduced test taking anxiety and greater growth in reading comprehension compared to the control group. Together, these findings help to build our burgeoning understanding of socioemotional competence—specifically self-efficacy—and academic outcomes in adolescence.

The current study also suggests that adolescents’ socioemotional resilience may have a mediating effect on various risk factors, including pandemic-related distress. This finding dovetails nicely with prior work suggesting that resilience (operationalized in terms of individuals’ faith in their ability to adapt to changing circumstances) similarly mediates the association between COVID-19-related stress and acute stress disorder symptoms among Chinese college students (Ye et al., 2020). We also found that both EF and resilience mediated the effect of participants’ mental health risk factors on reading. Among Chinese adolescents, the impact of stressful life events on school adjustment was serially mediated by both EF and resilience (operationalized in terms of feelings of personal competence and self-acceptance) (Zhang et al., 2019).

Our findings are also novel in drawing direct associations between risk factors, resilience factors, and reading outcomes as a specific metric of academic achievement. Importantly, the strongest indicators of our resilience factor were self-efficacy, social awareness, and self-management; post-hoc correlations revealed specific associations between self-efficacy, self-management, and reading skills. Both self-efficacy and self-management may be understood in the context of motivation research and theories of self-regulated learning. Self-management may be closely related to self-monitoring, a critical stage of self-reflection in the self-regulated learning cycle, and could potentially be associated with meta-cognitive strategy use relevant to reading success (Joseph & Eveleigh, 2011). Self-management, and socioemotional resilience more broadly, may also help students to sustain and control their attention to academic topics in the context of reading-related challenges present in LBLD and/or environmental risk factors in the COVID-19 context.

### Broader impacts for students with language based learning disabilities

The current findings demonstrate that even during a global pandemic, socio-emotional resilience may be associated with reading achievement among high risk learners. Reading difficulties for students with LBLD are chronic and persistent into adulthood; indeed, students in the current study demonstrate reading performance in the low-average and below average range. While resilience does not reduce or eliminate this reading difficulty, it may extend students’ bandwidth for tolerating related challenges, facilitate problem-solving, reduce the impact of stigma, temper the socioemotional consequences of learning difficulties, and/or foster an empathetic route for better understanding one’s self and others in the context of struggle. While we focus here on the student’s capacity to adapt to the environment, we also acknowledge the importance of responsive educators and environments for supporting student progress.

Efforts to address resilience have spanned research to practice (e.g., educational programs and interventions). Resilience interventions frequently focus on mindset and/or grit in younger children (e.g., Al Otaiba et al., 2022), and tend to query the impact of resilience on mental health or well-being as the primary proximal target outcome. This body of work has rarely been extended to examine the impact of resilience interventions on distal associations with academic achievement (however, see Hossain et al., 2021a, 2021b).

Our study suggests that self-efficacy and self-management are the two aspects of resilience most closely associated with reading outcomes in high-risk adolescent learners. This finding is novel, as most prior work has examined resilience in terms of grit and growth mindset in young children. However, we may draw insight from motivational interventions. A meta-analysis specific to reading self-efficacy (Unrau et al., 2018) suggests that (a) motivational interventions can successfully influence students’ self-efficacy beliefs, and (b) that self-efficacy and reading comprehension skill are positively related, although the directionality is unknown. Notably, however, effect sizes were somewhat larger for typical readers than struggling readers, and only one study in the meta-analysis was conducted with high schoolers. Future work should continue extending efforts to older students, and consider including self-efficacy and self-management dimensions in educational programming. Resilience-based training will be important to deliver in conjunction with reading interventions so that empowerment comes from both skill advancement as well as from training to self-advocate, navigate resources, and counter negative feedback (internal or external). Future work can clarify the association between self-efficacy measured generally for a student compared to self-efficacy specific to reading, as the current study used a general measurement. Additional efforts can also include co-designing research with students with LBLD and their educators and parents, building on efforts in the education and health domains (Dahlstrom-Hakki et al., 2021).

### Limitations

The current study was limited in several ways. The LBLD sample did not come from public schools; they were all immersed in a school specifically serving students with learning disabilities, with a specialized curriculum that emphasizes resilience. Because we lack a control group in a public school context, we are unable to determine whether participants’ resilience may have been elevated, or particularly powerful in overcoming risk, reducing the generalizability of the findings. The available data on participants did not include information on socioeconomic status, specific diagnoses, or duration at the school, which would have been valuable factors to consider. As LBLD is an umbrella term that captures various types of disabilities, there is untapped heterogeneity in the sample that may be related to reading outcomes.

We also lack objective information about the extent to which participants in the current study were impacted by COVID-19 over the school year (i.e., familial financial strain, illness or death of a loved one) as opposed to students’ *perceived* impact of COVID-19, operationalized in terms of their concern about the pandemic. Notably, however, prior research with adolescents has revealed negative psychosocial impacts of COVID-19, even in communities that were relatively less affected by the pandemic (De France et al., 2022).

Finally, we note a few important methodological considerations. The correlations between the individual indicators of resilience and reading measures are quite limited; only the bivariate correlations between self-efficacy and the T1 oral reading measures reach statistical significance. In our latent measurement model, factors reflect clusters of subscales from the same measure (e.g., all BRIEF indices clustered together). Although the organization of latent factors is logical and consistent with prior research, it is nevertheless possible that this model reflects differences in measurement rather than underlying psychological constructs. As we lack data on the concurrent validity of the resilience measures, additional research is needed to ensure that these constructs are being captured as intended. Due to our sample size, we used a data reduction strategy in combination with path analysis rather than a full structural model with latent factors.

Finally, our path models control for Time 1 single word reading, but do not fully control for oral passage reading skill at Time 1. Time 1 word reading was selected as a control variable because it is a highly correlated, lower-level skill that supports passage reading, accounting for much but not all of the individual difference in end-of-year oral passage reading. As such, our study does not inform questions about growth in students’ oral reading ability, but rather attempts to clarify the associations between risk, resilience, and reading over the course of an unprecedentedly challenging academic year. Nevertheless, the present findings represent an important step towards a deeper understanding of resilience factors that may support reading among older adolescents with learning disabilities, an understudied population.

## Conclusion

The current study examined the associations between risk and resilience factors and reading performance among adolescents with LBLD during the COVID-19 pandemic. We found that participants’ socioemotional risk, resilience, and self-regulation were all associated with students’ perceived COVID-19 impact at Time 1. However, risk and self-regulation deficits were not directly associated with oral reading composite scores at Time 2. Instead, socio-emotional resilience significantly predicted oral reading composite scores, and buffered the associations between COVID-related stress, mental health risk, self-regulation, and achievement. These findings add to a growing body of research focusing on reading skill in adolescents, particularly those with language-based learning disabilities, and point to the possible protective nature of socioemotional resilience in attenuating the impact of risk factors.

## Supplementary Information

Below is the link to the electronic supplementary material.

Supplementary file1 (DOCX 30 KB)
