# Supplementary material for: Risk and resilience correlates of reading among adolescents with language-based learning disabilities during COVID-19
Source: Read Writ. 2022 Nov 11;36(2):401–28. doi: 10.1007/s11145-022-10361-8 (PMC9649401; doi:10.1007/s11145-022-10361-8)
Supplement: Supplementary file 1 — Supplementary file1 (DOCX 30 KB) [file 11145_2022_10361_MOESM1_ESM.docx]

**Supplemental Materials**

Table S1

*Frequency statistics for BRIEF-2 executive function indices*

|  | Not elevated | Mildly elevated | Potentially clinically elevated | Clinically elevated |
| --- | --- | --- | --- | --- |
| Behavior regulation | 75 | 5 | 10 | 3 |
| Cognitive regulation | 64 | 10 | 9 | 10 |
| Emotion regulation | 65 | 15 | 8 | 5 |

Table S2

*Standardized path estimates for Model A predicting T2 GORT-5 Oral Reading Quotient*

| *Direct Effects* | | *Estimate* | *S.E.* | *p* |
| --- | --- | --- | --- | --- |
| Perceived COVID impact (*R*^2^ = .17) regressed on: | |  |  |  |
|  | Risk | .13 | .16 | .402 |
|  | Socio-emotional resilience | .40 | .12 | .001 |
|  | Self-regulation deficits | .36 | .16 | .025 |
| Self-regulation deficits (*R*^2^ = .57) regressed on: | |  |  |  |
|  | Risk | .46 | .09 | <.001 |
|  | Socio-emotional resilience | -.56 | .14 | <.001 |
| GORT-5 Oral Reading Quotient (*R*^2^ = .35) regressed on: | |  |  |  |
|  | Self-regulation deficits | .12 | .13 | .345 |
|  | Risk | .02 | .09 | .816 |
|  | Socio-emotional resilience | .26 | .13 | .005 |
|  | Perceived COVID impact | .05 | .10 | .623 |
|  | Age | -.24 | .07 | .001 |
|  | T1 word reading | .52 | .06 | <.001 |

Table S3

*Standardized path estimates for Model B predicting T2 GORT-5 Oral Reading Quotient*

| *Direct Effects* | | *Estimate* | *S.E.* | *p* |
| --- | --- | --- | --- | --- |
| Perceived COVID impact (*R*^2^ = .08) regressed on: | |  |  |  |
|  | Risk | .28 | .13 | .027 |
| Self-regulation deficits (*R*^2^ = .27) regressed on: | |  |  |  |
|  | Risk | .50 | .11 | <.001 |
|  | Perceived COVID impact | .05 | .13 | .691 |
| Socio-emotional resilience (*R*^2^ = .48) regressed on: | |  |  |  |
|  | Self-regulation deficits | -.77 | .12 | <.001 |
|  | Risk | .22 | .10 | .029 |
|  | Perceived COVID impact | .25 | .07 | <.001 |
| GORT-5 Oral Reading Quotient (*R*^2^ = .36) regressed on: | |  |  |  |
|  | Self-regulation deficits | .12 | .13 | .353 |
|  | Risk | .02 | .09 | .816 |
|  | Socio-emotional resilience | .26 | .13 | .005 |
|  | Perceived COVID impact | .05 | .10 | .623 |
|  | Age | -.24 | .07 | .001 |
|  | T1 word reading | .51 | .06 | <.001 |
| *Significant Indirect Effects* | | *Estimate* | *S.E.* | *p* |
| T1 perceived COVID impact to T2 GORT-5 Oral Reading through: | | | | |
|  | Resilience | .06 | .03 | .043 |
| T1 self-regulation deficits to T2 GORT-5 Oral Reading through: | | | | |
|  | Resilience | -.20 | .07 | .007 |
| T1 mental health risk to T2 GORT-5 Oral Reading through: | | | | |
|  | Self-regulation 🡪 Resilience | -.10 | .04 | .016 |

Table S4

*Standardized path estimates for Supplemental Model A predicting T2 GORT-5 reading comprehension*

| *Direct Effects* | | *Estimate* | *S.E.* | *p* |
| --- | --- | --- | --- | --- |
| Perceived COVID impact (*R*^2^ = .17) regressed on: | |  |  |  |
|  | Risk | .13 | .16 | .402 |
|  | Socio-emotional resilience | .40 | .12 | .001 |
|  | Self-regulation deficits | .36 | .16 | .025 |
| Self-regulation deficits (*R*^2^ = .57) regressed on: | |  |  |  |
|  | Risk | .46 | .09 | <.001 |
|  | Socio-emotional resilience | -.56 | .14 | <.001 |
| GORT-5 reading comprehension (*R*^2^ = .25) regressed on: | | | | |
|  | Self-regulation deficits | .08 | .13 | .526 |
|  | Risk | -.01 | .10 | .934 |
|  | Socio-emotional resilience | .23 | .10 | .018 |
|  | Perceived COVID impact | .07 | .10 | .473 |
|  | Age | -.21 | .08 | .013 |
|  | T1 word reading | .42 | .08 | <.001 |

Table S5

*Standardized path estimates for Supplemental Model B predicting T2 GORT-5 reading comprehension*

| *Direct Effects* | | *Estimate* | *S.E.* | *p* |
| --- | --- | --- | --- | --- |
| Perceived COVID impact (*R*^2^ = .08) regressed on: | |  |  |  |
|  | Risk | .28 | .13 | .027 |
| Self-regulation deficits (*R*^2^ = .27) regressed on: | |  |  |  |
|  | Risk | .50 | .11 | <.001 |
|  | Perceived COVID impact | .05 | .13 | .691 |
| Socio-emotional resilience (*R*^2^ = .48) regressed on: | |  |  |  |
|  | Self-regulation deficits | -.77 | .12 | <.001 |
|  | Risk | .22 | .10 | .029 |
|  | Perceived COVID impact | .25 | .07 | <.001 |
| GORT-5 reading comprehension (*R*^2^ = .26) regressed on: | |  |  |  |
|  | Self-regulation deficits | .08 | 13 | .529 |
|  | Risk | -.01 | .10 | .934 |
|  | Socio-emotional resilience | .23 | .09 | .015 |
|  | Perceived COVID impact | .07 | .10 | .473 |
|  | Age | -.21 | .08 | .013 |
|  | T1 word reading | .42 | .08 | <.001 |
| *Significant Indirect Effects* | | *Estimate* | *S.E.* | *p* |
| T1 perceived COVID impact to T2 GORT-5 Oral Reading through: | | | | |
|  | Resilience | .15 | .07 | .045 |
| T1 self-regulation deficits to T2 GORT-5 Oral Reading through: | | | | |
|  | Resilience | -.06 | .03 | .022 |
| T1 mental health risk to T2 GORT-5 Oral Reading through: | | | | |
|  | Self-regulation 🡪 Resilience | -.05 | .02 | .032 |
